# Paraoxonase 1 and Non-Alcoholic Fatty Liver Disease: A Meta-Analysis

**DOI:** 10.3390/molecules26082323

**Published:** 2021-04-16

**Authors:** Kazuhiko Kotani, Jun Watanabe, Kouichi Miura, Alejandro Gugliucci

**Affiliations:** 1 Division of Community and Family Medicine, Jichi Medical University, Tochigi 329-0498, Japan; m06105jw@jichi.ac.jp; 2 Division of Gastroenterology, Department of Medicine, Jichi Medical University, Tochigi 329-0498, Japan; miura385@jichi.ac.jp; 3 Glycation, Oxidation and Disease Laboratory, Touro University-California, Vallejo, CA 94592, USA; alejandro.gugliucci@tu.edu

**Keywords:** arylesterase, NAFLD, NASH, PON1, paraoxonase

## Abstract

Oxidative stress is involved in the pathophysiology of nonalcoholic fatty liver disease (NAFLD). However, reliable biomarkers of NAFLD in relation to oxidative stress are not available. While paraoxonase 1 (PON1) is an antioxidant biomarker, there appears to be mixed data on PON-1 in patients with NAFLD. The aim of this meta-analysis was to assess the current data on PON1 activity (i.e., paraoxonase and arylesterase) in patients with NAFLD. A PubMed, CENTRAL, and Embase search identified 12 eligible articles. In the meta-analysis, the paraoxonase activity was low in patients with NAFLD (mean difference (MD) −27.17 U/L; 95% confidence interval (CI) −37.31 to −17.03). No difference was noted in the arylesterase activity (MD 2.45 U/L; 95% CI −39.83 to 44.74). In a subgroup analysis, the paraoxonase activity was low in biopsy-proven nonalcoholic steatohepatitis (MD −92.11 U/L; 95% CI −115.11 to −69.11), while the activity in NAFLD as diagnosed by ultrasonography or laboratory data was similar (MD −2.91 U/L; 95% CI −11.63 to 5.80) to that of non-NAFLD. In summary, the PON1, especially paraoxonase, activity could be a useful biomarker of NAFLD. Further studies are warranted to ascertain the relevance of PON1 measurements in patients with NAFLD.

## 1. Introduction

Nonalcoholic fatty liver disease (NAFLD) is receiving a great deal of attention as a common cause of chronic liver disease [1]. In addition, nonalcoholic steatohepatitis (NASH), an advanced form of NAFLD, can progress to end-stage liver disorders, including liver cirrhosis and hepatocellular carcinoma [1]. Of note, NAFLD increases the risk of other comorbidities, including cardiometabolic diseases [2]. Accordingly, biomarkers that reflect the pathophysiology of NAFLD and the comorbidities are necessary to manage patients with NAFLD.

**Figure 1:**
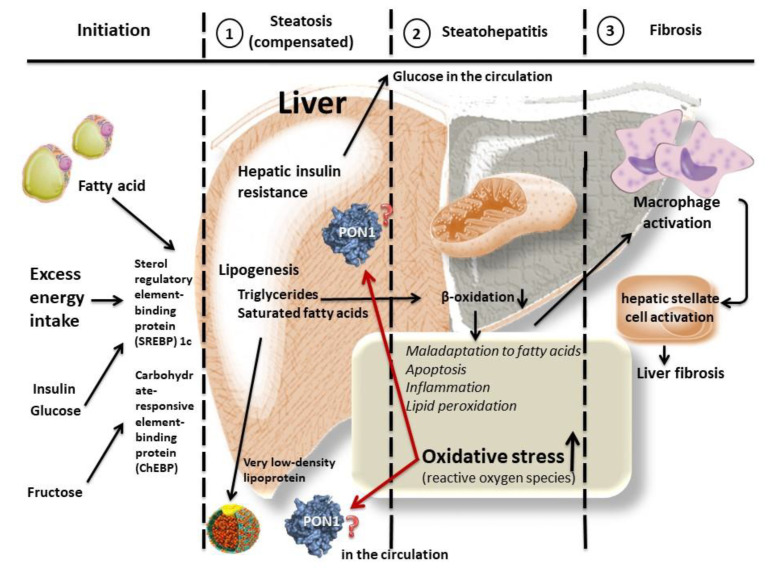
Speculative association of paraoxonase 1 (PON1) with nonalcoholic fatty liver disease (NAFLD). There are different grades of NAFLD, including steatohepatitis. Various molecules are associated with the development of the disease, and oxidative stress is a key to its progression. Antioxidants have a defensive role against the excess oxidative stress. Paraoxonase 1 (PON1), an antioxidant molecule, is produced in the liver and is secreted into the circulation; therefore, PON1 may play a relevant role in the pathophysiology of NAFLD. Circulating PON1 levels could serve as surrogate biomarkers of the underlying cause of the disease.

Oxidative stress is recognized as a causative factor of NAFLD, resulting in inflammation and fibrosis in the liver [3,4,5]. Although various molecules, lipids, and proteins are oxidized in that process, the detailed mechanisms remain to be fully elucidated [3,4,5]. Antioxidant factors can play a defensive role against excess oxidative stress in NAFLD [4,5]. Thus, biomarkers of oxidative stress/antioxidants may become useful for assessing and profiling NAFLD (Figure 1); however, there are currently no reliable biomarkers of NAFLD in relation to oxidative stress [6,7,8,9,10].

Paraoxonase 1 (PON1: aryldialkylphosphatase (EC3.1.8.1)) is known as an esterase molecule associated with apolipoprotein A-I and clusterin, which circulates in a high-density lipoprotein (HDL) particle [11]. PON1 hydrolyzes organophosphate compounds such as paraoxon and aromatic carboxylic acid esters such as phenylacetate, which functions as a paraoxonase and arylesterase [11]. PON1 is a key molecule with the antioxidant activity of HDL (as shown in vitro in animal and human studies) [12]. The antioxidant functions of PON1 are well-documented in pathways against atherosclerosis as follows [13,14]: (a) PON1 hydrolyzes peroxides and lactones in low-density lipoprotein (LDL) and HDL particles, as well as protects macrophages from oxidation; (b) it is a homocysteine-thiolactonase, which is posited as one of its physiological activities, (c) it is a xenobiotic metabolizer, (d) it protects beta cells, and finally, (e) it regulates endothelial homeostasis. In addition, PON1 acts on homoserine lactones, which are quorum-sensing molecules used by bacteria to prevent their growth, and participants in innate immunity [15,16].

Several studies have provided persuasive arguments for the role of PON1 in cardiovascular context in clinical settings [17,18]. PON1 is thought to be involved in the pathophysiology of various diseases, including kidney failure, neurological disorders, diabetes, and sleep apnea [19,20,21,22]. As PON1, which has detoxification activity, also hydrolyzes the metabolites of organophosphorus insecticides, the metabolites of organophosphorus pesticides as stemmed from PON1 are a marker of environmental exposure [23].

As PON1 is mainly produced in the liver [6,17], PON1 may become an important and useful research target in liver diseases. Earlier human studies demonstrated that the PON1 activity is low in patients with chronic liver diseases, including viral hepatitis and alcoholic liver injury [6,24]. A recent experimental study also demonstrated a low PON1 activity in rats with NAFLD [25]. However, it appears that there were mixed data on the PON1 activity levels in patients with NAFLD. Therefore, we aimed to determine, via meta-analyses, whether or not the PON1 activity in NAFLD was low in order to confirm the importance of PON1 measurements as a co-adjuvant in the diagnosis and prognosis of NAFLD.

## 2. Results

Table 1 shows a summary of the eligible studies [26,27,28,29,30,31,32,33,34,35,36,37]. The methods used to diagnose NAFLD varied: four studies used ultrasonography [28,29,30,35], two used laboratory data on the fatty liver index [36,37], and five used biopsies to prove NASH [26,27,31,33,34]. One study did not describe the diagnostic methodology [34]. Among the 12 studies, 12 measured the paraoxonase activity [26,27,28,29,30,31], and two also measured the arylesterase activity [29,31].

**Figure 2:**
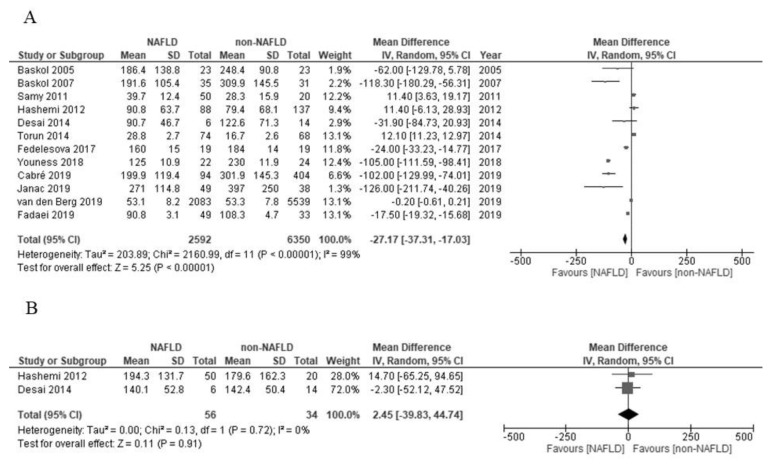
(**A**) Forest plot of the paraoxonase activity and nonalcoholic fatty liver disease. (**B**) Forest plot of the arylesterase activity and nonalcoholic fatty liver disease.

In the present meta-analysis, the paraoxonase activity among the patients with NAFLD was significantly low in comparison to those with non-NAFLD (mean difference (MD) –27.17 U/L; 95% confidence interval (CI) –37.31 to –17.03; I^2^ = 99%; Figure 2A). On the other hand, the arylesterase activity of the patients with NAFLD and non-NAFLD did not differ to a statistically significant extent (MD 2.45 U/L; 95% CI –39.83 to 44.74; I^2^ = 0%; Figure 2B).

**Figure 3:**
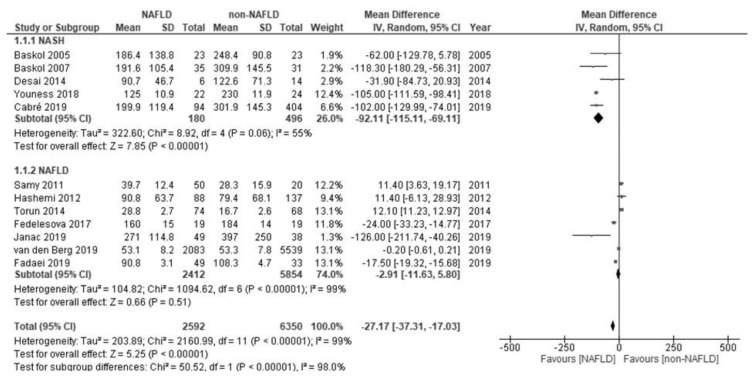
Forest plot of the paraoxonase activity and biopsy-proven nonalcoholic steatohepatitis (NASH; upper plot) and in nonalcoholic fatty liver disease (NAFLD), as diagnosed based on ultrasonography or laboratory data (lower plot).

Considering the overall low level of paraoxonase in patients with NAFLD, while the low activity was reported in five studies [26,27,31,33,34] and unchanged activity was reported in seven studies [28,29,30,32,35,36,37], we subsequently performed a sub-analysis of the studies on the paraoxonase activity. The paraoxonase activity was low in biopsy-proven NASH (MD –92.11 U/L; 95% CI –115.11 to –69.11), while the activity in NAFLD as diagnosed by ultrasonography or laboratory data was similar (MD –2.91 U/L; 95% CI –11.63 to 5.80) to that of non-NAFLD. The subgroup analysis according to the method used to diagnose NAFLD revealed a significant difference (biopsy-proven NASH versus NAFLD, as diagnosed by ultrasonography or laboratory data; *p* < 0.00001; Figure 3).

## 3. Discussion

The present meta-analyses revealed that the paraoxonase activity was significantly low and that the arylesterase activity was unaltered in patients with NAFLD in comparison to those with non-NAFLD. The paraoxonase activity was also observed to be significantly low in biopsy-proven NASH. These findings indicated that the PON1 activity, especially paraoxonase, may be a useful biomarker when studying the pathophysiology of NAFLD.

Liver diseases, including NAFLD, may lead to enhanced catabolism and/or the inactivation of PON1 molecules [24], which is a potential explanation for the low PON1 activity, as observed in the present meta-analysis. Furthermore, several possible reasons are considered for the differences in the results in relation to paraoxonase and arylesterase in NAFLD, which were observed in the present meta-analyses. PON1 is a promiscuous esterase, and its activity can be assessed using a variety of substrates, including paraoxon (paraoxonase activity) and phenylacetate (arylesterase activity). The substrates used in the PON1 measurement can elicit different behaviors of the PON1 species [17,18]. In addition, genetic polymorphisms of PON1 may be a factor related to these different activities [38,39]. The PON1-Q192R polymorphism influences the efficacy with which PON1 inhibits LDL oxidation, with the Q isoform being the most efficient and the R isoform being the least efficient [38], and arylesterase shows little variation, while the paraoxonase activity varies with the phenotype. However, the PON1 activity, which represents the integral genetic and acquired influences, is a much stronger determinant of the cardiovascular risk than the influence of related genetic polymorphisms on the activity [6,37,39]. The present meta-analysis included only two studies on arylesterase [29,31], and neither Q192R nor the other polymorphisms were studied. Thus, additional investigations are required to clarify the mechanistic role of paraoxonase versus arylesterase activity in patients with NAFLD.

In addition, since the PON1 levels may also be measured using other substrates (e.g., lactones), as its lactonase activity is presumably more physiological, further studies that measure the lactonase activities can provide different insight into the understanding of the pathophysiology of NAFLD in comparison to studies measuring the paraoxonase and arylesterase [23]. No studies comparing the various PON1 activity levels have been carried out in patients with NAFLD. This should also be considered for the PON1 levels to be used as biomarkers.

Although the use of PON1 protein levels as biomarkers may be envisioned, one study showed dissociated results—that is, the PON1 protein levels increased as the liver diseases progressed, even though the PON1 activity decreased, pointing to the production of faulty molecules and/or their inactivation in the circulation [24]. A study also reported that the PON1 mRNA and protein levels in the liver were increased in NASH [31]. One may posit that these increased PON1 mRNA and protein levels may reflect a compensatory production of PON1 after the onset of liver disease. However, another study reported that the production of PON1 was suppressed in liver disease, leading to low PON1 activity [6]. A combination assay of the PON1 protein concentration and activity may be warranted to understand their interaction and for both to be used as biomarkers of NAFLD.

Liver fibrosis and steatosis are detected in NASH, an advanced form of NAFLD. The management of NASH is, therefore, an issue. A study found that paraoxonase activity is inversely correlated with hepatic fibrosis in particular [24]. The present subgroup analysis also revealed that the paraoxonase activity was low in biopsy-proven NASH, not NAFLD, as diagnosed based on ultrasonography or laboratory data. The findings may be partly explained by the fact that NAFLD is a wide spectrum of liver pathologies (ranging from noninflammatory to inflammatory steatosis), and NAFLD as diagnosed based on ultrasonography or laboratory data could actually include patients with a wide pathological spectrum while a biopsy provides direct evidence of NASH. The biopsy is, however, an invasive procedure. Recently, several noninvasive methods (e.g., blood fibrosis markers and elastography) have become available for the diagnosis and staging of NAFLD [40,41]. Thus, paraoxonase activity, which can be determined noninvasively, may be a piece of information that can add to the other markers of NAFLD/NASH (rather than the biopsy) in the clinical management of NAFLD.

We must acknowledge some limitations to the present meta-analyses. First, the number of articles that were eligible for inclusion in the present review was limited. Second, the number of patients with biopsy-proven NASH was small. Furthermore, the reference range of PON1 measurements still has not been formally determined.

## 4. Materials and Methods

**Figure 4:**
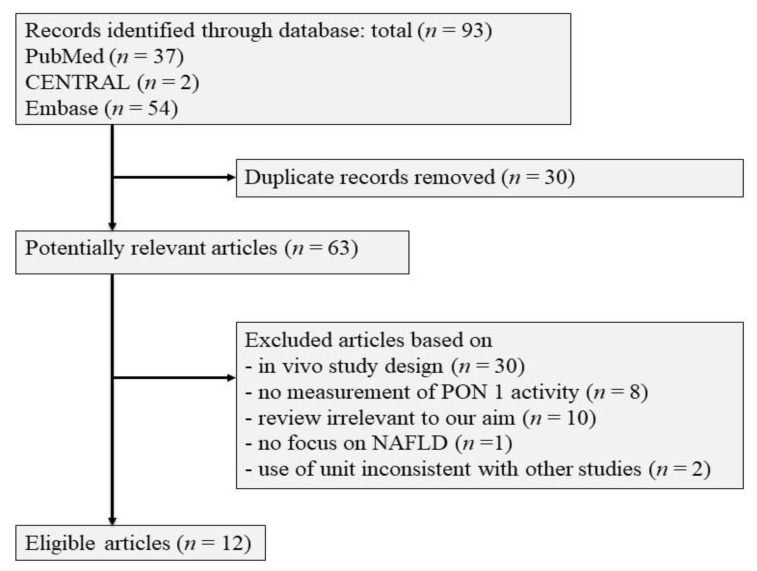
Flow chart of the selection of eligible studies.

A search of the PubMed, CENTRAL, and Embase databases was conducted using generic terms (“aryldialkylphosphatase (arylesterase)” (MeSH Terms) OR (All Fields) OR “paraoxonase” (All Fields)) AND (“steatohepatitis” (All Fields) OR “fatty liver disease” (All Fields)) for the literature published up to April 2021. After duplicate records were removed, sixty-three articles were found to be potential matches. After the search was limited to original articles on human studies and studies written in English, 50 articles were excluded. We analyzed the remaining 14 articles that reported PON1 activity levels in patients with and without NAFLD [26,27,28,29,30,31,32,33,34,35,36,37,42,43]. Two studies measured paraoxonase activity but were not included in the meta-analysis, because the units were unconventional [42,43]. Figure 4 demonstrates the flow in the selection of the 12 studies that were finally eligible for inclusion.

Furthermore, based on the eligible articles, random-effects meta-analyses of the PON1 activity among patients were performed using the generic inverse variance method in Review Manager 5.4.1 (RevMan 2020) [44]. The MD and 95% CI of the paraoxonase and arylesterase activity were calculated. When heterogeneity was seen (I^2^ statistic > 50%), the possible source of the heterogeneity was explored in a subgroup analysis [44], and the diagnostic method (e.g., biopsy-proven NASH and NAFLD as diagnosed based on ultrasonography or laboratory data) was considered in this study.

## 5. Conclusions

The present meta-analyses demonstrated that the PON1, especially paraoxonase, activity was low in patients with NAFLD. The low paraoxonase activity was also observed in biopsy-proven NASH. The paraoxonase activity could be a useful biomarker of NAFLD. On the other hand, further studies are warranted to ascertain the relevance of the PON1 measurements in patients with NAFLD. Particularly, studies to see the association between PON1 activity and the stage of NAFLD or longitudinal cohort and intervention studies with the PON1 measurements will be required in patients with NAFLD. In the measurements, measuring PON1 with an array of substrates is recommended.

## Figures and Tables

**Table 1:** Summary of the reviewed articles on NAFLD, including NASH, that investigated the PON1 activity.

Authors (Reference)	Age	Gender	Diagnosis	Activity in NAFLD (U/L)	Activity in Non-NAFLD (U/L)	Additional Notes
**Paraoxonase**						
Baskol et al. [26]	40 years (mean)	Men/women: 9/14	NASH; biopsy (histology)	186.4 ± 138.8	248.4 ± 90.8	Serum PON1 does not always correspond to the grade of NASH.
Baskol et al. [27]	39 years (mean)	Men/women: 22/13	NASH; biopsy (histology)	191.6 ± 105.4	309.9 ± 145.5	
Samy et al. [28]	47 years (mean)	Men/women: 22/28	NAFLD; ultrasonography	39.7 ± 12.4	28.3 ± 15.9	Statin treatment increases serum PON1.
Hashemi et al. [29]	40 years (mean)	Men/women: 50/33	NAFLD; ultrasonography	90.8 ± 63.7	79.4 ± 68.1	
Torun et al. [30]	About 13 years	Men/women: 26/83	NAFLD; ultrasonography	28.8 ± 2.7	16.7 ± 2.6	
Desai et al. [31]	12–18 years	Men/women: 4/2	NASH; biopsy (histology)	90.7 ± 46.7	122.6 ± 71.3	PON1 mRNA and protein levels in liver increase in NASH.
Fedelesova et al. [32]	Not detailed	Total 19 (gender: not detailed)	NAFLD; not detailed	160 ± 15	184 ± 14	
Youness et al. [33]	46 years (mean)	Men/women: 12/10	NASH; biopsy (histology)	125.0 ± 10.9	230.0 ± 11.9	
Cabré et al. [34]	46 years (mean)	Men/women: 25/69	NASH; biopsy (histology)	199.9 ± 119.4	301.9 ± 145.3	
Fadaei et al. [35]	51 years (median)	Total 49 (gender: not detailed)	NAFLD; ultrasonography	90.8 ± 3.1	108.3 ± 4.7	
Janac et al. [36]	48 years (mean)	Men/women: 16/33	NAFLD; the fatty liver index	271 ± 114.8	397 ± 250.0	
van den Berg et al. [37]	54 years (mean)	Men/women: 1422/661	NAFLD; the fatty liver index	53.1 ± 8.15	53.3 ± 7.78	
**Arylesterase**						
Hashemi et al. [29]	40 years (mean)	Men/women: 50/33	NAFLD; ultrasonography	194.3 ± 131.7	179.6 ± 162.3	
Desai et al. [31]	12–18 years	Men/women: 4/2	NASH; biopsy (histology)	140.1 ± 52.8	142.4 ± 50.4	

NAFLD, nonalcoholic fatty liver disease; NASH, nonalcoholic steatohepatitis; and PON1, paraoxonase 1.

## Data Availability

Data is contained within the present paper.

## Abbreviations

NAFLD	nonalcoholic fatty liver disease
NASH	nonalcoholic steatohepatitis
PON1	paraoxonase 1
